# rs2267531, a promoter SNP within glypican-3 gene in the X chromosome, is associated with hepatocellular carcinoma in Egyptians

**DOI:** 10.1038/s41598-019-43376-3

**Published:** 2019-05-03

**Authors:** Tarek Mohamed Kamal Motawi, Nermin Abdel Hamid Sadik, Dina Sabry, Nancy Nabil Shahin, Sally Atef Fahim

**Affiliations:** 1 Biochemistry Department, Faculty of Pharmacy, Cairo University, Cairo, Egypt; 2 Medical Biochemistry and Molecular Biology, Faculty of Medicine, Cairo University, Cairo, Egypt; 3 Biochemistry Department, Faculty of Pharmacy, Ahram Canadian University, Cairo, Egypt

**Keywords:** Cancer genetics, Genetic association study

## Abstract

Hepatocellular carcinoma (HCC) is a major health concern in Egypt owing to the high prevalence of hepatitis C virus (HCV) infection. HCC incidence is characterized by obvious male predominance, yet the molecular mechanisms behind this gender bias are still unidentified. Functional variations in X-linked genes have more impact on males than females. Glypican-3 (GPC3) gene, located in the Xq26 region, has lately emerged as being potentially implicated in hepatocellular carcinogenesis. The current study was designed to examine the association of −784 G/C single nucleotide polymorphism (SNP) in GPC3 promoter region (rs2267531) with HCC susceptibility in male and female Egyptian HCV patients. Our results revealed a significant association between GPC3 and HCC risk in both males and females, evidenced by higher C allele and CC/C genotype frequencies in HCC patients when compared to controls. However, no such association was found when comparing HCV patients to controls. Moreover, GPC3 gene and protein expression levels were significantly higher in CC/C than in GG/G genotype carriers in males and females. The CC/C genotype exhibited a significant shorter overall survival than GG/G genotype in HCC patients. In conclusion, GPC3 rs2267531 on the X chromosome is significantly associated with HCC, but not with HCV infection, in the Egyptian population.

## Introduction

Hepatitis C virus (HCV) infection is a major underlying cause of hepatic diseases globally, chronically infecting almost 150 million people. Egypt bears the highest HCV prevalence in the world reaching 14.7% of the population^1^. Although the infection is declining in developed countries, mortality due to cirrhosis and hepatocellular carcinoma (HCC) is still a major concern in Egypt. HCV-infected patients have 17-folds increased risk of HCC^2^. About 60–80% of HCV infection leads to chronic hepatitis in the patients, and 10–20% of those patients develop cirrhosis within 20–30 years. About 1–5% of patients with cirrhotic liver might develop HCC^3^.

HCC is the fifth most prevalent cancer globally and the third contributor to cancer-related death with about 500000 new cases annually. HCC accounts for almost three quarters of all hepatic cancers^4^. The mortality due to HCC is very high, remarkably in patients diagnosed in late-stages due to the lack of symptoms during early stages. This problem is more obvious in developing countries due to poor screening tools^5^. It was reported that there are associations between genomic alterations and clinical features including the clinical stage and the tumor size in HCC patients^6^. There is a demand for better understanding of the molecular mechanisms underlying the initiation and progression of HCC for earlier diagnosis and developing more effective therapeutic strategies^7^.

HCC exhibits a pronounced gender bias. Almost 85% of the cases occur in men as the male-to-female incidence ratio of HCC varies between 2–4:1 across different populations^8–10^. Functional variations in X-linked genes are likely to have more impact on males than on females^11^. It could, therefore, be reasonable to assume that certain genes located on the X chromosome may show an important role in the vulnerability to HCC.

Glypican-3 (GPC3) gene, located in the Xq26 region, encodes a membrane-bound heparan sulfate proteoglycan and significantly participates in cellular proliferation, migration and differentiation^12^. GPC3 regulates both stimulatory and inhibitory signals so it can either act as a tumor suppressor or an oncogenic protein and therefore plays a key role in regulating cancer cell growth. GPC3 is normally expressed in fetal and placental tissues^13^, but its expression in adults is limited to some organs including ovary, breast and lung where it acts as a tumor suppressor in these tissues. GPC3 expression is downregulated in ovarian and breast cancer^14,15^. On the other hand, in tissues with no expression in adults, GPC3 acts as an oncogenic protein as in HCC and Wilms tumor^16^. GPC3 is overexpressed in most HCCs but not in cirrhotic liver or in benign hepatic lesions^17,18^. It promotes the growth of HCC by increasing c-Myc expression, a usual target of the canonical Wnt signaling pathway^19^. Moreover, GPC3 disrupts cell proliferation by increasing resistance to apoptosis through dysregulating Bax, Bcl-2, cytochrome c and caspase-3 signaling. Several studies proposed that GPC3 would be a valuable serological and immunohistochemical marker for HCC^20,21^.

The promoter region of a gene regulates its expression due to binding of the transcription factors to specific nucleotide sequences in this region, subsequently affecting translation. Therefore, the gene promoter region is a critical determinant of an individual’s susceptibility to many diseases including cancer^22^. Single nucleotide polymorphism (SNP) in the promoter region of GPC3 is expected to modify the binding affinity of the promoter to transcription factors thereby modulating gene expression.

To the best of our knowledge, the association of GPC3 gene polymorphism with HCC risk has not been studied yet, although the association of GPC3 expression with HCC has been reported^21^. Therefore, our study aimed to explore whether −784 G/C gene polymorphism (rs2267531) in the promoter region of GPC3 in males and females is associated with HCC risk in HCV Egyptian patients.

## Results

### Demographic and clinical features of patients and controls

The demographic profile of HCV and HCC patients and controls enrolled in the study is described in Table 1. Age and sex did not significantly vary among the studied groups.

**Table 1:** Demographic characteristics and laboratory parameters in the HCV, HCC patients and healthy control.

Variables	Control (n = 126)	HCV (n = 130)	HCC (n = 110)	*P*-value
Age (years)	48.51 ± 18.5	48.25 ± 13.18	51.6 ± 10.82	0.15
Gender Male Female	66 (52.4%) 60 (47.6%)	78 (60%) 52 (40%)	60 (54.5%) 50 (45.5%)	0.45
Hemoglobin	10.84 ± 1.12	13.88 ± 1.48^a^	12.43 ± 1.71^ab^	<0.0001
WBCs (×10^3^)	4.12 ± 1.12	6.53 ± 1.99^a^	5.63 ± 2.2^ab^	<0.0001
Platelets (×10^3^)	186.4 ± 75.9	237 ± 110.2^a^	132.1 ± 58.66^ab^	<0.0001
T Bil (mg/dl)	0.9 ± 0.23	0.77 ± 0.3	1.27 ± 0.62^ab^	<0.0001
D Bil (mg/dl)	0.22 ± 0.13	0.38 ± 0.25^a^	0.53 ± 0.35^ab^	<0.0001
AST (U/L)	26.98 ± 6.41	54.24 ± 36.19^a^	73.17 ± 41.02^ab^	<0.0001
ALT (U/L)	27.52 ± 6.6	46.79 ± 23.89^a^	61.72 ± 37.67^ab^	<0.0001
ALP (U/L)	78.00 ± 29.17	128.6 ± 69.96^a^	183.9 ± 56.1^ab^	<0.0001
Albumin (g/dl)	4.13 ± 0.58	4.22 ± 0.42	3.32 ± 0.5^ab^	<0.0001
Creatinine (mg/dl)	0.97 ± 0.61	0.88 ± 0.21	0.86 ± 0.22	0.11
PC (%)	92.12 ± 5.8	79.26 ± 26.5^a^	75.74 ± 12.91^a^	<0.0001
PT-INR	1.1 ± 0.11	1.08 ± 0.11	1.25 ± 0.20^ab^	<0.0001
AFP (ng/ml)	3.3 (3.38–4.08)	2.7 (4.03–9.52)	202.5 (555.5–1187)^ab^	<0.0001

Data are expressed as mean ± SD, or n (%) or median (range).

Gender data were compared using Chi square (X^2^) test. The rest of the data were analyzed using one way ANOVA and Tukey’s multiple comparisons test.

^a^statistically significant from healthy controls.

^b^statistically significant from HCV.

WBCs, white blood cells; T Bil, total bilirubin; D Bil, direct bilirubin; AST, aspartate amino transferase; ALT, alanine aminotransferase; ALP, alkaline phosphatase; PC, prothrombin concentration; PT-INR,prothrombin time-international normalized ratios; AFP, alpha-fetoprotein.

### Hardy-Weinberg equilibrium (HWE), genotype distribution and allele frequencies of GPC3 rs2267531 polymorphism in patients and controls

As shown in Supplementary Table S1, a deviation from HWE was observed in the control, HCV and HCC groups.

The genotype distributions and allele frequencies of GPC3 rs2267531 polymorphism in the three studied groups are shown in Table 2. The CC/C genotype and C allele were significantly more prevalent in the HCC compared to either the HCV or control groups. The association of rs2267531 with HCV and HCC stratified by sex is demonstrated in Table 3. In female HCC patients, the rs2267531 CC genotype carriers exhibited a significantly more prevalent distribution than GG genotype compared to controls (OR = 5.71, *P* = 0.003) and to HCV patients (OR = 5.41, *P* = 0.008). Moreover, the C allele frequency was significantly higher in the female HCC patients compared to controls (OR = 2.51, *P* = 0.001), and to HCV patients (OR = 2.43, *P* = 0.002). In male HCC patients, C allele carriers exhibited a significantly higher frequency compared to controls (OR = 6.22, *P* = 0.000002) and to HCV patients (OR = 5.58, *P* = 0.000002).

**Table 2:** Frequency distribution for genotypes and alleles for GPC3 rs2267531 in patients and control groups.

	Control	HCV	HCC	
GG/G	64 (0.51)	68 (0.52)	28 (0.25)	
GC	37 (0.29)	33 (0.26)	15 (0.14)	
CC/C	25 (0.2)	29 (0.22)	67 (0.61)	
*P*-value	<0.0001^ł^	0.75^*^	<0.0001^#^	<0.0001^♦^
G	118 (0.63)	114 (0.63)	53 (0.33)	
C	69 (0.37)	68 (0.37)	107 (0.67)	
*P*-value	<0.0001^ł^	0.93^*^	<0.0001^#^	<0.0001^♦^

^ł^χ^2^ test for difference in the frequency distributions between HCC vs control.

^*^χ^2^ test for difference in the frequency distributions between HCV vs control.

^#^χ^2^ test for difference in the frequency distributions between HCC vs HCV.

^♦^χ^2^ test for difference in the frequency distributions in the study population.

**Table 3:** Association of SNP rs2267531 alleles and genotypes with HCC, HCV patients stratified by gender.

Cases/controls	Sex	G allele distribution N (%)	C allele distribution N (%)	Allele statistics OR (95% CI), *P*	p-BD for alleles	Genotype distribution, N (%)			Logistic regression statistics OR (95% CI), *P*	p-BD for genotype	Risk Ratio (95% CI)
						GG/G	GC	CC/C			
HCC	F	35 (35%)	65 (65%)	2.43^a,#^ (1.38–4.28), 0.002		10 (20%)	15 (30%)	25 (50%)	5.41^a,#^ (1.6–18.2), 0.008		2.26^a,#^ (1.13–4.53)
	M	18 (30%)	42 (70%)	5.58^b,#^ (2.67–11.65), 0.000002		18 (30%)		42 (70%)	5.58^b,#^ (2.67–11.65), 0.000002		2.37^b,#^ (1.62–3.47)
	Combined			3.31^c,#^ (2.13–5.17), 1.32 × 10^−7^	0.08				5.54^c,#^ (2.95–10.39), 9.67 × 10^−8^	0.97	2.36^c,#^ (1.69–3.28)
HCV	F	59 (56.7%)	45 (43.3%)	1.03^a,*^ (0.61–1.75), 1		13 (25%)	33 (63.5%)	6 (11.5%)	1.05^a,*^ (0.28–3.92), 1		1.04^a,*^ (0.42–2.56)
	M	55 (70.5%)	23 (29.5%)	1.11^b,*^ (0.54–2.31), 0.85		55 (70.5%)		23 (29.5%)	1.11^b,*^ (0.54–2.31), 0.85		1.08^b,*^ (0.64–1.82)
	Combined			1.06^c,*^ (0.69–1.63), 0.87	0.86				1.10^c,*^ (0.58–2.08), 0.89	0.94	1.06^c,*^ (0.69–1.67)
Control	F	69 (57.5%)	51 (42.5%)	2.51^a,ł^ (1.45–4.34), 0.001		16 (26.6%)	37 (61.7%)	7 (11.7%)	5.71^a,ł^ (1.8–18.08), 0.003		2.35^a,ł^ (1.22–4.51)
	M	48 (72.7%)	18 (27.3%)	6.22^b,ł^ (2.87–13.4), 0.000002		48 (72.7%)		18 (27.3%)	6.22^b,ł^ (2.87–13.48), 0.000002		2.57^b,ł^ (1.67–3.93)
	Combined			3.4^c,ł^ (2.18–5.3), 5.71 × 10^−8^	0.06				6.06^c,ł^ (3.18–11.51), 3.47 × 10^−8^	0.9	2.51^c,ł^ (1.76–3.59)
*P*-value			<0.0001					<0.0001			

^a^Females vs female, ^b^Males vs males, ^c^Combined vs combined.

^#^HCC vs HCV, ^*^HCV vs control, ^ł^HCC vs control.

p-BD: *P* value of Breslow-Day test for heterogeneity of the OR.

Combined *P* value and combined OR were calculated by Cochran-Mantel-Haenszel test with 1df.

HCC, hepatocellular carcinoma; HCV, hepatitis C virus; OR, odds ratio.

We found that the OR conferred by the rs2267531 was not significantly different between both sexes in each of the studied groups (*P* > *0*.*05*, Breslow-Day test for heterogeneity of OR). By combining males and females, rs2267531 CC/C genotype carriers exhibited significantly higher frequencies in HCC patients compared to controls (OR = 6.06, *P* < 0.0001) and to HCV patients (OR = 5.54, *P* < 0.0001). Similarly, combining males and females revealed a significantly higher C allele frequency in HCC patients versus controls (OR = 3.4, *P* < 0.0001), and HCV patients (OR = 3.31, *P* < 0.0001).

**Figure 1:**
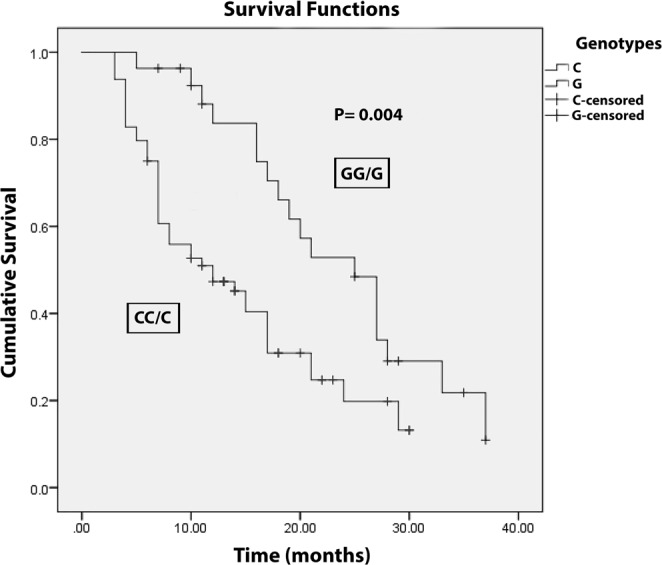
Kaplan-Meier and Log-rank survival curves for HCC patients in relation to GPC3 rs2267531 genotypes.

The aforementioned findings suggest a significant association of the GPC3 rs2267531 SNP with HCC risk in Egyptian patients. However, GPC3 rs2267531 SNP was not significantly associated with HCV risk and any of the clinicopathological parameters. On the other hand, Kaplan-Meier and Log-rank survival tests revealed shorter OS in GPC3 rs2267531 CC/C genotype compared with GG/G genotype in the HCC patients (see Fig. 1). The survival period was significantly longer in the GG/G genotype than in the CC/C genotype. The model for end-stage liver disease (MELD) score was non-significantly lower in the GG/G genotype than in the CC/C genotype (Supplementary Table S2). OS showed non-significant difference between males and females with *P*-value = 0.2. A significant moderate negative correlation was found between GPC3 protein levels and survival period in males only (Supplementary Fig. S1a).

### Comparison of gene and protein expression levels of GPC3 among the studied groups

**Figure 2:**
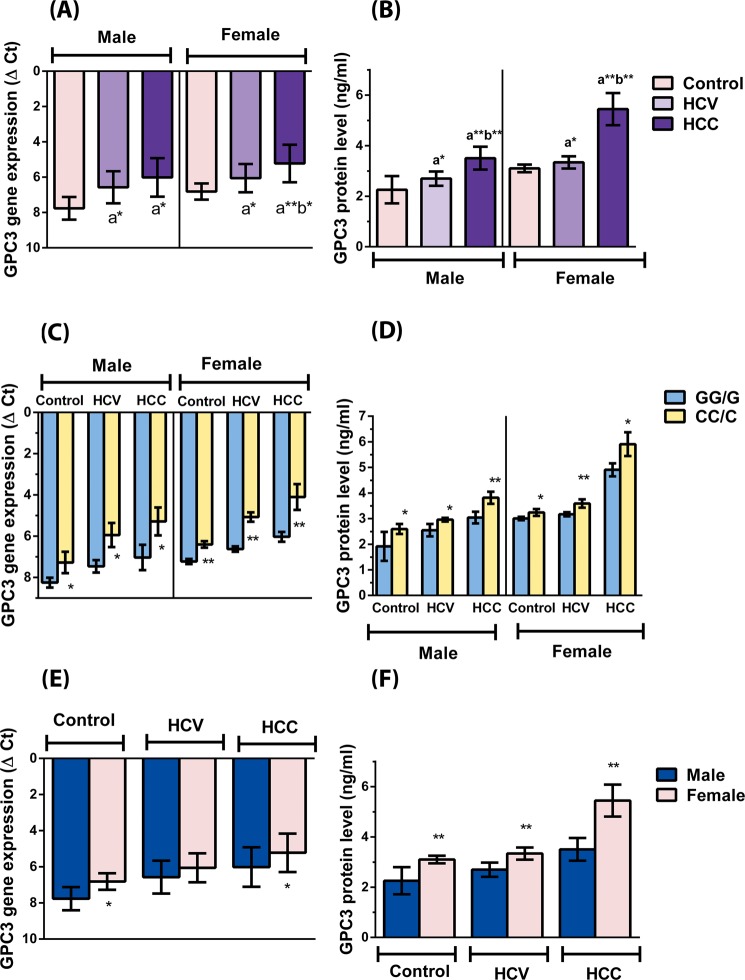
Gene and protein expression levels of GPC3 among the three studied groups (**A** and **B**), among the genotypes within each group (**C** and **D**) and among males and females within each group (**E** and **F**). Protein expression level is expressed as mean ± SD. Gene expression level is expressed as ΔCt mean ± SD, where ΔCt = Ct value of GPC3 - Ct value of β-actin, higher gene expression is equivalent to a smaller ΔCt value. The data were analyzed using Student’s t-test for comparing between 2 groups and one way ANOVA followed by Tukey’s multiple comparisons test for comparing between the three studied groups. ^a^Statistically significant from control, ^b^Statistically significant from HCV. *Indicates significance at p < 0.05. **Indicates significance at p < 0.0001.

The gene expression level of GPC3 was significantly higher in HCV and HCC patients in both males and females when compared to the corresponding controls. Moreover, GPC3 gene expression level was significantly higher in HCC than in HCV females, but was only apparently higher in HCC than in HCV males where the difference did not reach statistical significance (see Fig. 2A).

The GPC3 protein level was significantly higher in the HCV and the HCC patients compared to the controls in males and females. Moreover, GPC3 protein expression level was significantly higher in the HCC males and females when compared to their corresponding HCV counterparts (see Fig. 2B).

Comparing GPC3 expression among rs2267531 genotypes, a significant association was detected between GPC3 rs2267531 polymorphism and its gene and protein expression. The CC/C carriers had significantly higher GPC3 gene and protein expression levels compared to the GG/G carriers in the three studied groups as shown in Fig. 2C,D, respectively.

Comparing GPC3 expression levels based on gender revealed a significantly higher protein expression in females than in males in the three studied groups. An analogous pattern of variation was noted in the gene expression levels being only apparent in the HCV group, but reaching statistical significance in the controls and HCC patients (see Fig. 2E,F).

Western blot analysis confirmed the significantly higher protein expression in HCC patients compared to HCV patients and controls, as well as in HCV patients compared to the controls (see Supplementary Fig. S2).

### Serum α-fetoprotein (AFP) levels, correlation with GPC3 protein expression and ROC analysis

Serum AFP level was significantly higher in HCC patients compared to either controls or HCV patients (Table 1). As shown in Supplementary Fig. S1b, there was a weak positive correlation between AFP and GPC3 protein levels (r = 0.21).

The discriminatory power analysis of gene and protein expression levels of GPC3 for differentiation of HCC patients versus non-HCC subjects was assessed using a ROC curve as shown in Supplementary Table S3. The results of the ROC curve analysis suggest that the protein level of AFP has better diagnostic accuracy than GPC3

### Sanger sequencing

**Figure 3:**
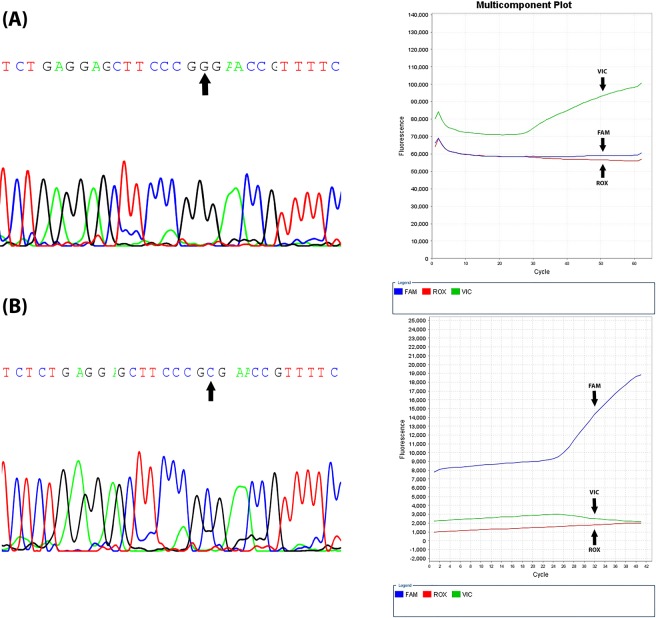
A Sanger sequencing chromatogram generated by ABI Genetic Analyzer showing GPC3 DNA sequence (5′ to 3′) and multicomponent plot generated through PCR showing the wild-type (**A**) and mutant-type (**B**) in GPC3 −784 upstream in the promoter region.

By comparing the results of TaqMan allelic discrimination method with Sanger sequencing, the gold standard method in clinical diagnosis, a perfect accordance occurred between both methods for all genotypes in the tested samples (see Fig. 3).

## Discussion

HCV infection, for which Egypt bears the highest incidence in the world, is a major risk factor for the development of HCC. The present study explored the association between rs2267531 SNP in the promoter region of GPC3 and the risk of HCV-related HCC in Egyptians.

Although the X chromosome is an intriguing target for association studies, the number of reported disease-associated loci in this chromosome is very low^23^, possibly due to the challenge of selecting powerful statistical tests of association^24^.

The current study revealed for the first time the association of GPC3 rs2267531, a new locus on chromosome Xq26, with HCC risk. Our findings are in accordance with the notion that a mutation in GPC3 gene may contribute to X-linked recessive inheritance causing the phenotype HCC to be more expressed in males than in females^25^. Mutated GPC3 in Simpson-Golabi-Behmel syndrome results in tissue overgrowth and an increased risk of embryonic malignancies in males, while female carriers may have mild manifestations^26^. GPC3 rs2267531 (−784 upstream in the promoter region) is located within the 2 kb at the 5′ UTR of GPC3 gene. This region has been reported to include the elements that establish a minimal promoter, as concluded from the locations of the binding sites for transcription factors^27^.

By using the allelic discrimination method that was confirmed by direct DNA sequencing, we examined −784 G/C gene polymorphism (rs2267531) in the promoter region of GPC3. Our results showed that the observed genotype frequencies of the GPC3 rs2267531 polymorphism did not agree with those expected for the HWE in the control, HCV and HCC groups. The deviation from HWE in the case group may be indicative of association with the disease^28,29^. The deviation of the control group from HWE could be attributed to the extensively mixed Egyptian population. In fact, control samples that arise from a combination of genetically different subpopulations may not be in HWE^30^. In this study, Egyptians had a higher frequency of the heterogeneous genotype GC than the protective GG. Several genetic studies reported a high degree of genetic heterogeneity in the Egyptian population, suggesting that this population is descendant from an African, Asian and Arabian mixture^31^. Waples^32^ suggested that females and males who have the same allele frequencies will have genotypic frequencies in HWE. If otherwise (as observed in our study), the homogametic gender will generally show an excess of heterozygotes.

According to the NCBI map database, the incidence of SNP rs2267531 in different ethnic populations revealed G and C allele frequencies of 0.4897 and 0.5103 in African Carribbeans, 0.49 and 0.51 in Europeans, 0.6974 and 0.3026 in Japanese, and 0.66 and 0.33 in Han Chinese, respectively. In the current study, the G and C allele frequencies in the control group were 0.63 and 0.37, respectively which are quite analogous to those observed in the Japanese and Han Chinese populations having G as the major allele. Obviously, there is ethnicity-related variation in the incidence of rs2267531 polymorphism. This further explains the observed deviation of the controls from HWE.

There was a significant difference among the three studied groups concerning SNP rs2267531 distribution at both allelic and genotype levels. GPC3 −784 CC/C genotype and C allele frequencies were significantly higher in HCC patients in comparison with HCV patients and controls, suggesting a protective effect of the G allele. Moreover, we found a significant association between HCC risk and the rs2267531 C allele both in the allelic and genotypic comparisons by logistic regression analyses. The high OR indicates the strength of association between the studied locus and the disease. This may be due to the SNP up-regulatory effect on GPC3 expression, being found in the CG**C**G DNA sequence that is a typical recognition sequence for many transcription elements as AtSR/CaMTA that are regulated by Ca^2+^/calmodulin via binding various regulating kinases and transcription elements^33^. This assumption is supported by the significant association observed between the rs2267531 CC/C genotype and the high gene and protein expression of GPC3. Several studies showed that promoter region SNPs are related to high gene expression^34,35^.

In agreement with former studies^17,18^, GPC3 gene and protein expression levels were significantly higher in HCC patients compared with HCV patients and controls. Moreover, HCV had significantly higher GPC3 gene and protein expression levels than controls. This is in agreement with former study in a Japanese population reporting that GPC3 expression was observed more frequently in patients with HCV infection and with early HCC than controls^36^. Moreover, two studies in Egypt have reported that patients with chronic HCV had elevated levels of serum GPC3 compared to healthy controls^37,38^. On the other hand, another Egyptian study reported that GPC3 is a specific marker for HCC patients and does not increase in HCV infected patients^39^. Our observation of higher GPC3 expression in females than in males in the three studied groups could be explained on the basis that 15–25% of X-linked genes may escape X chromosome inactivation at least to some degree so women can still express many genes from their inactive X chromosomes. On average, 5.1% of X-linked genes showed higher expression in females compared to males^40^.

Serum AFP showed significantly higher levels in HCC patients when compared to either HCV patients or controls. ROC analysis revealed that the sensitivity of GPC3 in the HCC group was lower than that of AFP at the same specificity when compared to the control and non-HCC groups. In contrast, GPC3 was reported to have higher sensitivity than AFP in distinguishing HCC from benign liver disease and early detection of HCC in Egyptian patients^37^. Our study demonstrated a significant but weak correlation between AFP level and GPC3 protein expression. On the contrary, another study reported no correlation between them^41^.

No significant correlation was detected between the studied SNP variants and clinical parameters such as liver enzymes, albumin, bilirubin, prothrombin, creatinine, RBCs, WBCs and platelets. However, Kaplan-Meier and Log-rank survival tests indicated significantly shorter OS in rs2267531 CC/C compared with GG/G genotype carriers in HCC patients. In the same context, our results revealed a significantly longer survival period in GG/G than in CC/C genotype carriers. A previous study reported notably shorter OS and disease-free survival in patients with overexpressed GPC3 in HCC tissues^42^. Moreover, GPC3-positive HCC patients had a significantly lower 5-year survival rate than those who were GPC3-negative^43^. In the current study, the MELD score was apparently, but non-significantly, lower in GG/G than in CC/C genotype carriers. It was reported that there was no correlation between GPC3 expression and MELD score^44^. Although higher GPC3 expression in our study has been associated with shorter OS in HCC males and females collectively, OS did not vary significantly between males and females. Moreover, the survival time was negatively correlated with GPC3 protein level in males only. The lack of correlation between GPC3 expression and HCC outcome in females could be attributed to the protective role of the sex steroid hormone estrogen in HCC^45,46^. This explanation could be supported by reports of significantly higher incidence of liver tumor in response to ovariectomy and/or testosterone supplement^47,48^.

In conclusion, the present study suggests that GPC3 rs2267531 may be associated with the risk of HCC, but not with HCV infection, in the studied sample of the Egyptian population. Specifically, GPC3 rs2267531 C allele frequency is associated with the risk of HCC. Furthermore, our data demonstrate that the CC/C genotype of GPC3 rs2267531 is associated with increased gene and protein expression of GPC3. Further studies on larger sample size, samples from other geographical regions and samples of other types of cancers are recommended to examine the associations between GPC3 −784 G/C polymorphism and cancer risk.

## Subjects and Methods

### Subjects

The study included 240 Egyptian patients comprising 130 patients diagnosed with HCV infection and 110 patients with HCV-dependent HCC. The patients were enrolled from the Endemic Medicine and Gastroenterology Department, Faculty of Medicine, Cairo University, during the period from June 2014 till October 2017.

All HCV patients were positive for HCV-RNA. The HCC patients had HCV infection that was detected by positive anti-HCV Ab. The HCC patients were identified by pathology, cytology, imaging (ultra-sound and computed tomography), and serum AFP level. Tumor characteristics (number and size of lesions, extrahepatic metastases, macroscopic vascular invasion and TNM stage), portal vein thrombosis, chest, brain, and total-body bone CT (to rule out extrahepatic metastases), and portal hypertension were also assessed. Child Pugh score and MELD score were also assessed. None of the HCC patients were previously treated by either chemotherapy or radiotherapy or had any other cancers.

A total of 126 volunteers matched to the patient population by sex and age were enrolled in this study as controls (Table 1). None of the volunteers had a previous history of hepatic disease; all had liver functions within the reference ranges, normal liver and biliary system ultrasound, and negative serological findings for autoimmune and viral hepatic diseases.

The Institutional Review Board at Faculty of Pharmacy, Cairo University approved the study (Permit number: BC 1813), and a written informed consent was obtained from all participants prior to the study. All procedures and methods were performed in accordance with the institution’s relevant guidelines and regulations of Helsinki-1975.

Subjects were excluded from the study if they had hepatitis B virus, human immunodeficiency virus antibodies, active schistosomiasis, diabetes or fatty liver disease, in addition to alcohol or heavy metal in blood.

### Blood sampling and laboratory assays

Approximately 10 ml of venous blood was collected from each participant and complete blood count was performed. A portion of blood was collected in EDTA tubes and stored at −80 °C until DNA and RNA were extracted for subsequent genetic analysis and RT-qPCR, respectively. Another portion was collected in citrate-containing vacutainers to separate plasma for the assay of albumin and prothrombin time-international normalized ratio (PT-INR). A third portion was collected in plain tubes to separate serum for estimation of ALT, AST and ALP activities, total and direct bilirubin, HCV-RNA, HCV specific antibody titers, AFP, and GPC3 levels. All samples were stored at −80 °C till use. The degree of cirrhosis in liver cancer was assessed according to the Child score classification. Patients were categorized into Child-Pugh grades A (5 to 6 points), B (7 to 9 points) and C (10 to 15 points).

### Primer and probe design

The sequence of GPC3 was obtained from NCBI database. Ensembl genome browser 90 was used to show all variants to prevent designing primers that overlie SNP sites. Then Primer3Plus was used to design the primers and probes specific for each allele. After all, Blast and MFEprimer-2.0 were used to check primer and probe specificity, dimers and hairpins. We selected a SNP (rs2267531) in the 5′UTR that should be expected to modify the binding affinity of the promoter to transcription factors, thereby regulating gene expression, and with MAF > 20%.

### GPC3 gene expression

Total RNA was isolated from the blood samples with a total RNA purification kit provided by Jena Bioscience (Munich, Germany) and stored at −80 °C. RNA was converted into its complementary DNA by cDNA archive kit (Applied Biosystems, Foster City, California, USA). qPCR was performed by using GoTaq PCR master mix (Promega Co., Madison, USA). A protocol that included an initial denaturation step at 95 °C for 10 minutes, followed by 40 cycles of denaturing at 95 °C for 15 s, annealing and extension at 60 °C for 1 minute then 60 °C for 30 s was performed on a 7500 Real-Time PCR System (Applied Biosystems, Foster City, California, USA). The following oligonucleotide primers were used: 5′-GTCCCTGAACGCGACTATTT-3′ (sense), 5′-AGCTTGTGCCAGCTCTTT-3′ (antisense) for GPC3, and 5′-ATGCTCTCCCTCACGCCATC-3′ (sense) and 5′-CAGGATTCCATACCCAAGA-3′ (antisense) for β-actin as an internal control.

### Genotyping of SNP rs2267531 in GPC3

DNA extraction from peripheral blood mononuclear cells was carried out using the Wizard^®^ Genomic DNA Purification Kit (Promega, Madison, USA) in accordance with the manufacturer’s guidelines. The genomic DNA was measured by using Nanodrop (Thermo Scientific, USA). The samples were stored at −20 °C till further use.

Genotyping was performed using qPCR with TaqMan^®^ allelic discrimination assay software (Applied Biosystems, Foster City, California, USA) using Applied Biosystems Step one plus 7500 qPCR System. Amplification was carried out at 95 °C for 10 minutes, followed by 40 cycles of 95 °C for 15 s and then 60 °C for 1 minute and 60 °C for 30 s for annealing and extension. The following primers and two labeled probes were used; primers: 5′-GTCCCTGAACGCGACTATTT-3′ (sense) and 5′-AGCTTGTGCCAGCTCTTT-3′ (antisense), probes: VIC-CCGGGAAGCTCCTCAGAGAGTAGAA, and FAM-GCGGGAAGCTCCTCAGAGAGTAGAA. The passive reference dye used was ROX.

### Sanger sequencing

In order to validate the TaqMan^®^ allelic discrimination method, 20 samples from each genotype were set for sequencing. In brief, PCR sequencing cycling reaction was conducted using a reverse primer then gel electrophoresis were applied to analyze the amplified yield. The sequencing cycling PCR products were purified then gel electrophoresis was repeated with these purified products as shown in Supplementary Fig. S1. Finally, the PCR products were sequenced with reverse primer using a Big Dye Terminator 3.1 Cycle Sequencing Kit (Applied Biosystems, Foster City, California, USA), according to the producer’s guidelines.

The reactions were executed in a computerized single capillary Sequencer; ABI PRISM 310 Genetic Analyzer (Applied Biosystems, Foster City, California, USA). The obtained sequences were analyzed via the GenBank BLAST tool to confirm the nucleotide sequence. Then, the sequences were edited and aligned by means of MEGA7 Sequence Alignment. The primers used were 5′-GTGGGATTTGGGTGTAGGATAC-3′ (sense) and 5′-AGCTTGTGCCAGCTCTTT-3′ (antisense) with an amplicon size of 506 bp.

### Determination of serum GPC3 levels

Serum GPC3 protein levels were measured using an ELISA kit provided by Bioassay Technology Laboratory (Shanghai, China) in compliance with its operational guidelines.

### Western blot

The serum of all the studied groups was lysed in NP-40 lysis buffer (50 mM Tris, pH 7.4; 150 mM NaCl; 1% NP-40). Proteins were separated from the whole cell lysate in a 12% SDS-PAGE. Then, the electrophoresed proteins were blotted onto Amersham™ Hybond® P Western blotting polyvinylidene fluoride membranes (GE10600021 Sigma, Sigma-Aldrich, MO, USA), and incubated with the primary antibodies; rabbit anti-GPC3 (1:500) at 4 °C overnight, and mouse anti-β-actin (1:1,000) (Santa Cruz Biotechnology, Santa Cruz, California, USA) at room temperature for 1 h. Incubation with sheep anti-mouse IgG- horseradish peroxidase (Amersham Pharmacia, United Kingdom) then followed. Quantification of the Western blot bands was performed by image analysis using the ChemiDoc MP imaging system (version 3) made by Bio-Rad (Hercules, California, USA). Relative density of each band was evaluated and normalized with β-actin.

### Statistical analysis

Variations between two groups were analyzed by Chi square test for categorical variables and Student’s t-test for numerical variables. To assess differences among the three groups, one-way ANOVA was employed with subsequent Tukey’s multiple comparisons post-hoc test. Sensitivity and specificity were calculated using the ROC analysis. Spearman’s correlation analysis was used to correlate between AFP and GPC3 protein levels. The ORs, Risk Ratio (RRs) and 95% CIs were estimated for association analysis by logistic regression. The Breslow-Day test was used to test for heterogeneity between ORs, and was calculated with the Cochran-Mantel-Haenszel (CMH) test. A *P*-value of less than 0.05 for a two-tailed test was considered statistically significant. Kaplan–Meier analysis and Log-rank survival tests were used to determine the overall survival (OS). Statistical analyses were accomplished using the GraphPad Prism 6 (GraphPad Software, California, USA) and SPSS software version 20.0 (SPSS Inc. Chicago, IL, USA). HWE was tested by R environment version 1.5.6 of the Hardy–Weinberg package. Chi square test results exceeding 5.99 (i.e. *P* < 0.05) indicated a deviation from HWE^49,50^.

## Supplementary information

Supplementary tables and figures

## Data Availability

The authors declare that all the data in this manuscript are available.
