# Supplementary material for: rs2267531, a promoter SNP within glypican-3 gene in the X chromosome, is associated with hepatocellular carcinoma in Egyptians
Source: Sci Rep. 2019 May 3;9:6868. doi: 10.1038/s41598-019-43376-3 (PMC6499880; doi:10.1038/s41598-019-43376-3)
Supplement: Supplementary file 1 — Supplementary tables and figures [file 41598_2019_43376_MOESM1_ESM.docx]

**rs2267531, a promoter SNP within glypican-3 gene in the X chromosome, is associated with hepatocellular carcinoma in Egyptians**

**Tarek Mohamed Kamal Motawi^1ǂ^, Nermin Abdel Hamid Sadik****^1ǂ^, Dina Sabry^2ǂ^, Nancy Nabil Shahin^1ǂ^ and Sally Atef Fahim^3*ǂ^**

**Supplementary Table S1. Hardy Weinberg equilibrium for GPC3 rs2267531 with or without males**

| **Group** | **Genotypes** | | | | | **X^2^ *P*-values** | |
| --- | --- | --- | --- | --- | --- | --- | --- |
|  | ***m*G** | ***m*C** | ***f*GG** | ***f*GC** | ***f*CC** | ***all*** | ***female*** |
| **Control** | 48 | 18 | 16 | 37 | 7 | 0.015 | 0.042 |
| **HCV** | 55 | 23 | 13 | 33 | 6 | 0.018 | 0.035 |
| **HCC** | 18 | 42 | 10 | 15 | 25 | 0.037 | 0.016 |

The Chi square test for Hardy-Weinberg equilibrium (HWE), using two degrees of freedom, was calculated in both sexes according to the following alleles and genotypes: in males, p and q are the number of males carrying the G and C allele, respectively, while in females, p^2^, 2pq and q^2^ are the number of females carrying one of the probable genotypes: GG, GC and CC, respectively.

**Supplementary Table S2. The associations of GPC3 -784 G/C SNP with the clinicopathological findings in HCC patients**

| **Genotype**  **Parameter** | **CC/C** | **GG/G** | ***P*-Value** |
| --- | --- | --- | --- |
| **Hemoglobin** | 12.46 ± 1.8 | 12.46 ± 1.67 | 0.99 |
| **WBCs (x10^3^)** | 5.4 ± 2.3 | 5.3 ± 2.4 | 0.83 |
| **Platelets (x10^3^)** | 128.9 ± 51.32 | 143.5 ± 84.71 | 0.31 |
| **T Bil (mg/dl)** | 1.28 ± 0.59 | 1.31 ± 0.73 | 0.85 |
| **D Bil (mg/dl)** | 0.53 ± 0.32 | 0.56 ± 0.44 | 0.73 |
| **AST (U/L)** | 78.36 ± 41.45 | 69.25 ± 47.39 | 0.35 |
| **ALT (U/L)** | 65.83 ± 41.04 | 58.93 ± 36.69 | 0.44 |
| **ALP (U/L)** | 183.2 ± 59.12 | 188.9 ± 58.6 | 0.69 |
| **Albumin (g/dl)** | 3.37 ± 0.47 | 3.2 ± 0.49 | 0.11 |
| **Creatinine (mg/dl)** | 0.84 ± 0.18 | 0.91 ± 0.29 | 0.19 |
| **PC (%)** | 74.22 ± 12.95 | 77.38 ± 13.81 | 0.29 |
| **PT-INR** | 1.27 ± 0.18 | 1.23 ± 0.29 | 0.37 |
| **Survival period in months** | 11.8 ± 7.3 | 20.74 ± 9.5 | < 0.0001 ** |
| **MELD score** | 10 (6-16) | 9.5 (6-18) | 0.36 |
| **Child–Pugh score** | *A*, n=43; *B*, n=21; *C*, n=3 | *A*, n=18; *B*, n=10; *C*, n=0 | 0.5 |

Data are expressed as mean ± SD or median (interquartile range), *P* <0.05 was significant. Data were compared using Student’s t-test for parametric tests, the Mann‐Whitney test for non-parametric tests and Chi square test for categorical variables.

WBCs, white blood cells; T Bil, total bilirubin; D Bil, direct bilirubin; AST, aspartate amino transferase; ALT, alanine aminotransferase; ALP, alkaline phosphatase; PC, prothrombin concentration; PT-INR, prothrombin time-international normalized ratios; MELD: model for end‐stage liver disease.

**Supplementary Table S3. Diagnostic accuracy of GPC3 and AFP by ROC curve**

| **Variable(s)** | **AUC** | ***P*-value** | **95% Confidence Interval** | | **Cut-off value** | **Sensitivity (%)** | **Specificity (%)** |
| --- | --- | --- | --- | --- | --- | --- | --- |
|  |  |  | **Lower Bound** | **Upper Bound** |  |  |  |
| GPC3 protein level (Control vs HCC) | 0.93 | <0.0001 | 0.84 | 0.98 | >3.46 ng/ml | 75.8 | 100 |
| GPC3 protein level (HCC vs non-HCC) | 0.89 | <0.0001 | 0.81 | 0.95 | >3.52 ng/ml | 75.8 | 94.8 |
| AFP protein level (Control vs HCC) | 0.97 | <0.0001 | 0.94 | 0.99 | >8.1ng/ml | 95.4 | 100 |
| AFP protein level(HCC vs non-HCC) | 0.96 | <0.0001 | 0.94 | 0.98 | >9.9 ng/ml | 93.5 | 94.5 |
| GPC3 gene expression (Control vs HCC) | 0.78 | <0.0001 | 0.64 | 0.88 | >2.13 | 75 | 75 |
| GPC3 gene expression (HCC vs non-HCC) | 0.68 | 0.004 | 0.56 | 0.79 | >2.13 | 75 | 51.52 |

Non-HCC, HCV patients and healthy controls; ROC, Receiver–operator curves.





**Supplementary Fig.S1. Spearman correlation analysis of GPC3 protein level and survival time in males (a) and AFP and GPC3 protein levels in HCC patients (b).**

**r: Spearman correlation coefficient**

*** Indicates statistical significance**


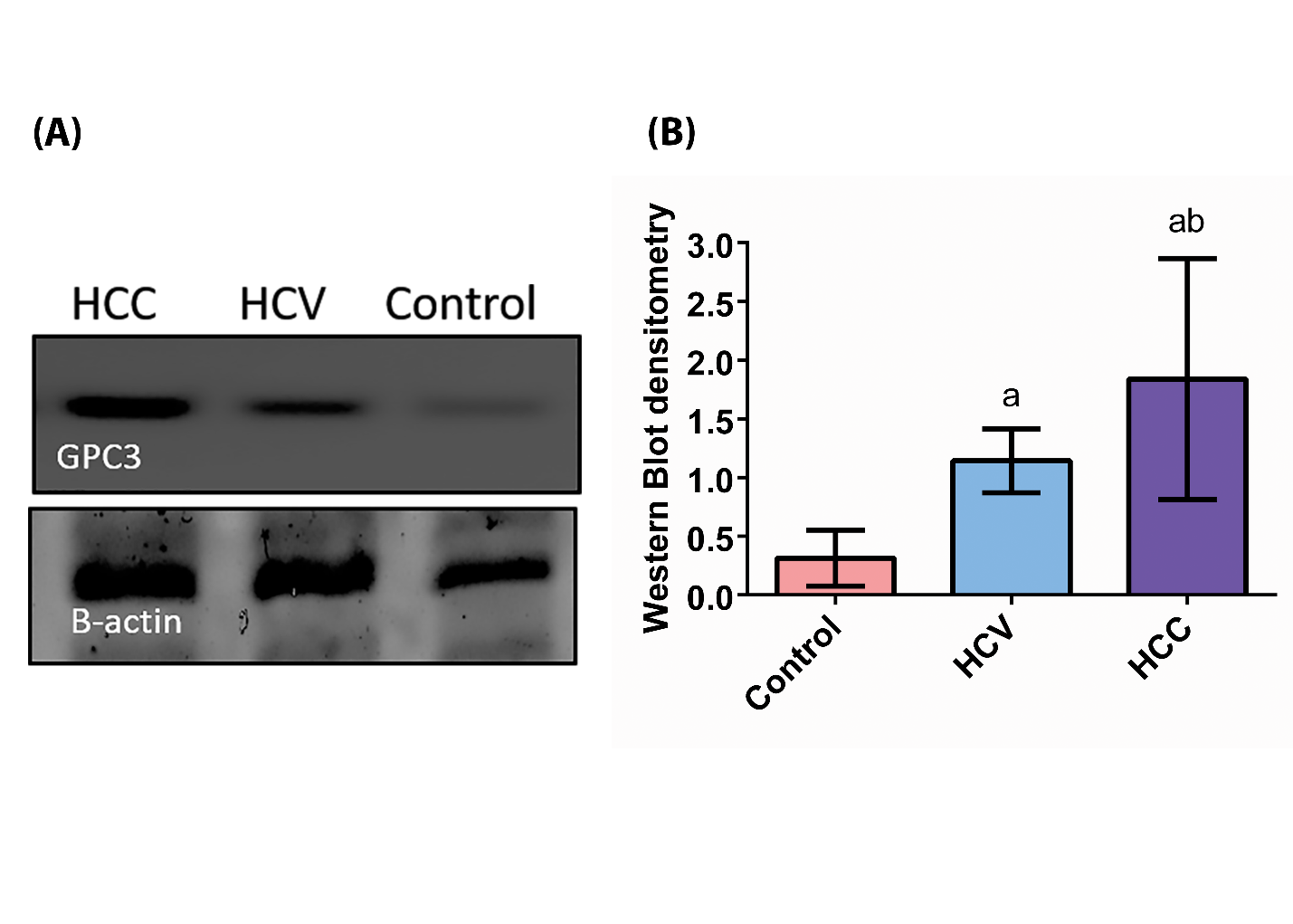


**Supplementary Fig.S2. Protein expression levels of GPC3 as analyzed by Western blotting (A) and relative quantification represented as mean** *±* **SD (B).**

The data were analyzed using one way ANOVA followed by Tukey's multiple comparisons test.

^a^ Statistically significant from control group at p <0.0001

^b^ Statistically significant from HCV at *P* <0.0001


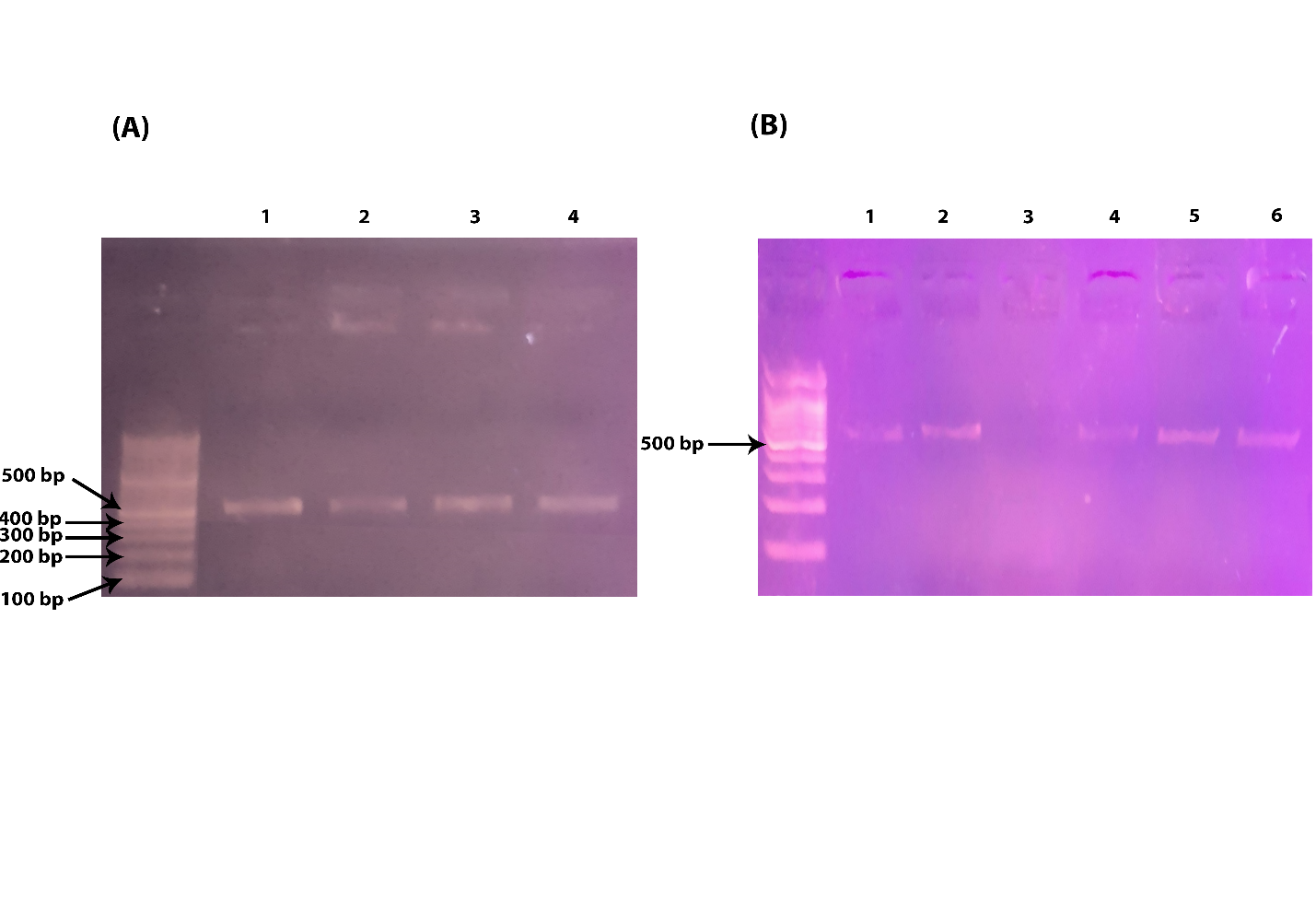


**Supplementary Fig.S3. 2% Agarose gel electrophoresis analysis of 506 bp PCR amplification products. The gel was stained with 0.5 μg/ml ethidium bromide. Lane 1 contains molecular marker (100-bp DNA step ladder) (A) Before PCR products purification, (B) After PCR products purification for subsequent DNA sequencing.**
